# Markers of Iron Status Are Associated with Risk of Hyperuricemia among Chinese Adults: Nationwide Population-Based Study

**DOI:** 10.3390/nu10020191

**Published:** 2018-02-09

**Authors:** Xiangping Li, Tingchao He, Kai Yu, Qian Lu, Rashad Alkasir, Guifang Guo, Yong Xue

**Affiliations:** 1 Peking University School of Nursing, No.38 Xueyuan Road, Haidian District, Beijing 100191, China; xiangping@bjmu.edu.cn (X.L.); luqian@bjmu.edu.cn (Q.L.); 2 Department of Nutrition & Food Hygiene, School of Public Health, Peking University Health Science Center, No.38 Xueyuan Road, Haidian District, Beijing 100191, China; hetingchao@bjmu.edu.cn (T.H.); Kai.Yu@rd.nestle.com (K.Y.); 3 CAS Key Laboratory of Pathogenic Microbiology and Immunology, Institute of Microbiology, Chinese Academy of Science, No.1 Beichen West Road, Chaoyang District, Beijing 100101, China; rashad.84@hotmail.com

**Keywords:** serum uric acid, serum ferritin, hyperuricemia, the China Health and Nutrition Survey, iron overload

## Abstract

Background: Elevated serum uric acid (SUA) involved in iron metabolism, has been increasingly recognized as a risk factor for gout and cardiovascular diseases. The objective of this study was to examine the associations between markers of iron status with risk of hyperuricemia (HU) in Chinese adult population. Methods: Data were extracted from the 2009 wave of the China Health and Nutrition Survey, consisting of 7946 apparently healthy adults. Serum ferritin (SF), transferrin, soluble transferrin receptors (sTfR), hemoglobin (Hb), high-sensitivity C-reactive protein (hs-CRP), and SUA were measured. Diet was assessed with three consecutive 24 h recalls. Demographic characteristics, smoking status, alcohol consumption, and physical activities were investigated using a structured questionnaire. Multilevel mixed-effects models were constructed to estimate the associations of SF, transferrin, sTfR, and Hb with SUA and the risk of HU. Results: The crude prevalence of HU was 16.1%. SF, transferrin, and Hb levels were positively associated with SUA and the risk of HU after adjustment for cluster effects and potential confounders (all *p*-trend < 0.05). Compared with participants in the lowest quartile of SF, those in the highest quartile had significantly higher SUA concentrations (β = 0.899 mg/dL, 95% confidence interval (CI): 0.788, 1.010; *p* < 0.001) and higher risk of HU (odds ratio (OR) = 3.086, 95% CI: 2.450, 3.888; *p* < 0.001). Participants with the highest quartile of transferrin had significantly higher SUA concentrations (β = 0.488 mg/dL, 95% CI: 0.389, 0.587; *p* < 0.001) and higher risk of HU (OR: 1.900; 95% CI: 1.579, 2.286; *p* < 0.001) when compared with those with the lowest quartile. In male participants, those in the highest quartile of Hb had significantly higher risk of HU when compared to the reference group (OR: 1.401, 95% CI: 1.104, 1.777; *p* < 0.01); however, this association was not found in female participants (OR: 1.093; 95% CI: 0.821, 1.455; *p* = 0.544). Conclusion: SF, transferrin, and Hb levels were positively associated with the risk of HU, and additional studies are needed to confirm the findings, as well as to elucidate their underlying mechanisms.

## 1. Introduction

Serum uric acid (SUA), the end product from purine degradation in humans and higher primates, has not been defined, but it can function as an antioxidant [1,2]. The accumulating epidemiologic data have demonstrated that hyperuricemia (HU) or elevated SUA levels are not only critical to gout [3], but also found to be potential pathogenic factors for some other chronic non-communicable diseases (NCDs), such as diabetes mellitus [4], cardiovascular diseases [5], nonalcoholic fatty liver disease [6], and cancer [7]. In the past few decades, the prevalence of HU has rapidly increased both in western countries and China [8,9]. Several risk factors of HU have been implicated, including older age, menopause, family history of NCDs, lack of physical activity, inadequate dietary intake, higher levels of inflammatory cytokines, such as interleukin-6 (IL-6), tumor necrosis factor alpha (TNF-α), and high-sensitivity C-reactive protein (hs-CRP), and some genetic variations [3,10,11,12,13,14].

Increasing evidence shows that body iron metabolism plays an important role in the development of HU [2,15,16,17,18]. Serum ferritin (SF) is the most widely used biomarker in clinical and epidemiological studies to evaluate body iron stores, despite being affected by inflammation status. Several studies [2,15,16,17] have assessed the relationship between SF and the risk of HU, but few studies have adjusted for some important lifestyle factors, such as cigarette smoking, physical activity, and dietary factors, which are associated with the risk of HU [11,12,19,20,21]. As such, it is still not clear whether the relationship between SF and the risk of HU is independent of these risk factors. Moreover, it is well known that the monosodium urate crystals, which are caused by increased SUA, can lead to inflammation in joints and surrounding tissues [13]. Previous studies showed that the concentrations of SF increase rapidly on exposure to trauma or infection [22], and remain high during the chronic phases of inflammatory process [23], so it is necessary to exclude those participants with acute inflammation, and adjust for the confounders of chronic inflammation. However, only a few studies had adjusted for the parameters of inflammation status, such as C-reactive protein (CRP) [15] or white blood cell count (WBC) [17]. Furthermore, the associations between other important proteins involved in iron metabolism, in addition to SF, such as transferrin and soluble transferrin receptors (sTfR), and SUA, have not been widely investigated. Hemoglobin (Hb) is the most commonly used hematological test to assess iron status, although some studies have demonstrated that Hb is not a good indicator of iron stores [24]. A previous study reported that Hb was significantly, positively correlated with the risk of HU [18]; however, it is still not clear whether this association between Hb and the risk of HU is independent of lifestyle factors and inflammation markers.

To date, no nationwide population-based studies have been conducted in China to explore the relationship between markers of body iron status and the risk of HU. Therefore, our nationwide population-based study, which regarded SF, transferrin, sTfR, and Hb as the primary exposures, with control for clustering of data at multiple levels (individual, household, community, and province), and adjustment for potential confounders, including age, gender, body mass index (BMI), dietary factors, physical activity, and inflammation status, was performed on a sample from a nationwide cross-sectional study. The purpose of the present study was to explore the aforementioned associations based on the following hypothesis: markers of iron status are associated with SUA concentrations and the risk of HU in the Chinese adult population.

## 2. Materials and Methods

### 2.1. Study Population

The data were extracted from the 2009 wave of the China Health and Nutrition Survey (CHNS), during which fasting blood collection and assessment were conducted for the first time. The CHNS, as an observational cohort study based on all community-dwelling subjects in 9 diverse provinces (Guangxi, Guizhou, Heilongjiang, Henan, Hubei, Hunan, Jiangsu, Liaoning, and Shandong) throughout China, aimed to understand the association between social and economic transformation of Chinese society, and the health and nutritional status of its population [25]. So far, there have been a total of nine waves of nutritional surveys, conducted in 1989, 1991, 1993, 1997, 2000, 2004, 2006, 2009, and 2011, respectively. For each wave, two cities in urban sites and four counties in rural sites were selected randomly in each selected province according to a weighted sampling scheme, and then primary sampling units (including urban and suburban neighborhoods within the selected cities, and villages and townships within the selected counties) were selected randomly. Twenty randomly selected households were surveyed within each unit, and all individuals within a household were interviewed. Details of the CHNS have been described elsewhere [25,26,27]. In the 2009 wave of CHNS, a total of 11,929 apparently healthy participants from 36 urban neighborhoods, 37 suburban neighborhoods, 37 towns, and 108 villages were enrolled. In the present study, our analyses were focused on the adult population aged 18 years and over (*n* = 10,081). We excluded 1434 participants without blood collection, 78 without blood assessments on SUA, SF, transferrin, sTfR, or Hb, 308 with acute inflammation (i.e., hs-CRP levels > 10 mg/L) [28,29], 4 with missing information on drinking and smoking, 150 without physical examination, 106 without assessing dietary intake, and 55 with pregnancy status, resulting in 7946 participants (3710 men and 4236 women) in the final analysis. All participants signed their written informed consent before participation in the study, and the research was reviewed and approved by the Institute Review Board of the University of North Carolina at Chapel Hill, and the National Institute of Nutrition and Food Safety, China Center for Disease Control and Prevention.

### 2.2. Laboratory Data

After at least 12 h of overnight fasting, a blood sample (12 mL) was collected by venipuncture in the morning. The blood sample (4 mL) was collected into a red-stoppered tube with separating gel, and was centrifuged 30 min after blood collection, at 3000× *g* for 15 min; serum samples were frozen and stored at −86 °C for later laboratory analysis. Another blood sample (500 μL) was collected into a lavender-stoppered tube with EDTA for routine blood examination. All samples were verified and analyzed in a national central lab in Beijing (medical laboratory accreditation certificate ISO 15189:2007) according to strict quality control standards [30,31,32]. SUA concentrations were measured with the use of an enzymatic colorimetric method on a Hitachi 7600 automated analyzer (Hitachi Inc., Tokyo, Japan) by determiner regents (Randox Laboratories Ltd., Crumlin, UK). SF concentrations were measured via radioimmunology on a gamma counter XH-6020 (North Institute of Bio-Tech, Beijing, China). Previous studies [28,29] have shown that hs-CRP > 10 mg/L is usually considered indicative of acute inflammatory processes, and an artificially higher SF concentration would be caused by inflammation, so we excluded those participants with an hs-CRP > 10 m/L, and multiplied SF values in the rest of the participants with an hs-CRP > 5 mg/L by 0.85 for male and 0.68 for female [33]. Serum transferrin and sTfR concentrations were both measured via nephelometry on a Siemens B-type natriuretic peptide assay (Siemens, Erlangen, Germany). Serum hs-CRP concentrations were measured with the use of a high-sensitivity immunoturbidimetric method on a Hitachi 7600 automated analyzer by determiner regents (Denka Seiken Co., Ltd., Niigata, Japan). Hb concentrations in whole blood were measured on a LH75 hematology analyzer (Beckman Coulter, Brea, CA, USA). The concentrations of SUA, SF, transferrin, sTfR, hs-CRP, and Hb measured in CHNS study have been used in previous studies [31,32,34,35,36,37]. HU was defined as SUA concentrations ≥ 7 mg/dL in men and ≥6 mg/dL in women [12,38].

### 2.3. Assessment of Dietary Intake

The dietary assessment in the CHNS was performed using a combination of three consecutive 24 h dietary recalls (including two weekdays and one weekend) at the individual level, and a food inventory weighting at the household level over the same three-day period. During one 24 h dietary assessment at household level, all foods and condiments remaining after the last meal of previous day, all foods purchased from markets or picked from gardens, food waste, as well as food inventory at the last meal of current day, were weighed using a digital kitchen scale with a minimum limit of 1 g and a maximum of 3 kg. Household food consumption on each day was determined by examining alterations of the inventory from the beginning to the end of the day. All the members of a household were asked to report all of the food consumed at home and away from home during the previous day, with the aid of food models and picture. The necessary information of each food consumed was collected and recorded by trained interviewers, including the types, amounts, types of meal, and locations of the meal. Individual amounts of food in each dish were estimated using the proportion of each dish consumed by each person combined with the household inventory. Detailed information of dietary data collection has been described elsewhere [26,39,40,41]. Dietary intakes of total energy, carbohydrate, protein, and fat, carbohydrate (E%, percentage of energy), protein (E%), and fat (E%) were calculated by CHNS based on means of the three 24 h recalls and the Chinese Food Composition Table 2004 and 2009 [42,43], and categorized into four groups according to their quartiles.

### 2.4. Assessment of Physical Activity and Other Covariates

Comprehensive data on physical activities were collected in the CHNS by staff-administered questionnaires, including four categories: domestic, occupational, transportation, and leisure physical activities. All activities were reported in average hours per week spent in the past year. The level of physical activity was assessed by using metabolic equivalent (MET)-hours-per-week, which was pooled by the total multiplications of time spent in each activity and specific MET value based on the Compendium of Physical Activities [44]. Using the measurement method of physical activity in the CHNS, a series of research findings of high quality have been published [26,45,46].

Measurement of weight without heavy clothes and shoes was taken by trained health workers, using a calibrated beam balance, to the nearest 0.1 kg. Measurement of height without shoes was taken using a portable SECA stadiometer to the nearest 0.1 cm. BMI was calculated as weight (kg) divided by height squared (m^2^). Other socioeconomic and lifestyle covariates, including age, gender, nationality, education, current smoking, and alcohol consumption, were measured with a structured questionnaire by trained and experienced interviewers.

### 2.5. Statistical Analyses

Before the progress of data analysis, Shapiro–Wilk test was used to determine whether characteristics of participants had a normal distribution or not. The characteristics of participants are presented as means ± standard deviation (SD), or medians (interquartile ranges) for quantitative variables with or without normal distribution, respectively, and percentages (n) for categorical variables, and were compared between participants with HU and the others by using Student’s *t* tests, Mann–Whitney U tests, or Chi-squared tests. Pearson’s correlations were explored to examine the correlations of SF, transferrin, and Hb with SUA after ln transformation of SF. To assess the associations of SF, transferrin, and Hb levels with SUA concentrations, the multilevel (four levels) mixed-effects linear regression models (SF, transferrin, and Hb levels) with individuals (level 1) nested within household (level 2), community (level 3), and province (level 4) were constructed using the statistical software MLwiN 2.36. Furthermore, multilevel mixed-effects logistic regression models were constructed to assess their associations with the risk of HU. β regression coefficients (and 95% confidence intervals (CIs)) in the linear regression and prospective odds ratios (ORs) (and 95% CIs) in the logistic regression were calculated for SUA and the risk of HU, respectively. We considered three sequential models in the aforementioned analyses: model 1 without any adjustments; model 2 adjusted for age, gender, BMI (<18.5 kg/m^2^, 18.5–24.9 kg/m^2^, 25.0–29.9 kg/m^2^, or ≥30 kg/m^2^), nationality (Han or others), education (≤6 years, 6.1–9.0 years, or >9 years), smoking status (current or not current), alcohol consumption (yes or no), and physical activity (quartile); model 3 adjusted as for model 2 plus total energy intake (quartile), protein intake (quartile), fat intake (quartile), carbohydrate (E%) (quartile), protein (E%) (quartile), fat (E%) (quartile), and inflammation status (hs-CRP levels < 1 mg/L, 1–3 mg/L, or 4–10 mg/L) [47]. Descriptive and correlation analyses were carried out using the Statistical Package for the Social Sciences (SPSS Inc., Chicago, IL, USA). All statistical tests were 2-sided, and *p* < 0.05 was considered statistically significant.

## 3. Results

Participant characteristics in the present study are displayed in Table 1. Compared with participants without HU, those with HU were more likely to belong to urban residents, be older, be male, have higher education, have higher BMI, smoke tobacco, consume alcohol, have lower levels of physical activity, and more likely to have higher intakes of total energy, protein, and fat. The percentages of energy intake from protein and fat were significantly higher, and the percentage of energy intake from carbohydrate was significantly lower in participants with HU when compared to the others. Remarkably, participants with HU had significantly higher concentrations of SF, transferrin, Hb, and hs-CRP when compared to those without HU. However, there were no significant differences in nationality, dietary carbohydrate intake, and sTfR levels between the two groups.

**Figure 1:**
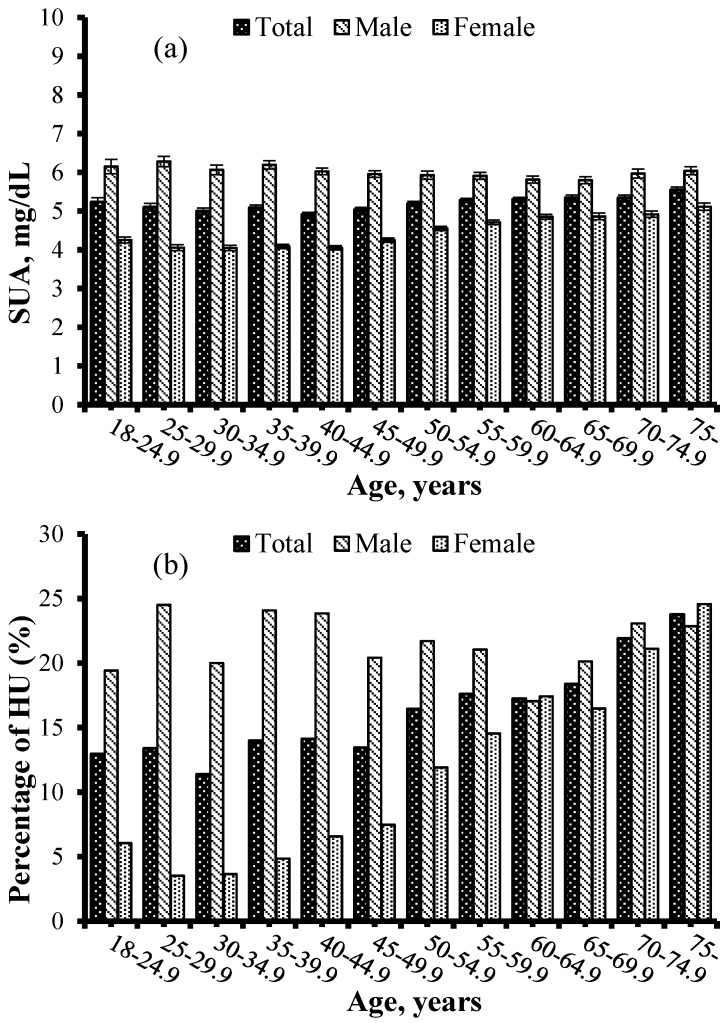
SUA concentrations (**a**) and crude prevalence of HU (**b**) for 7946 participants according to their age and gender. Data of SUA concentrations were expressed as means ± standard error (SE). SUA, serum uric acid; HU, hyperuricemia.

**Figure 2:**
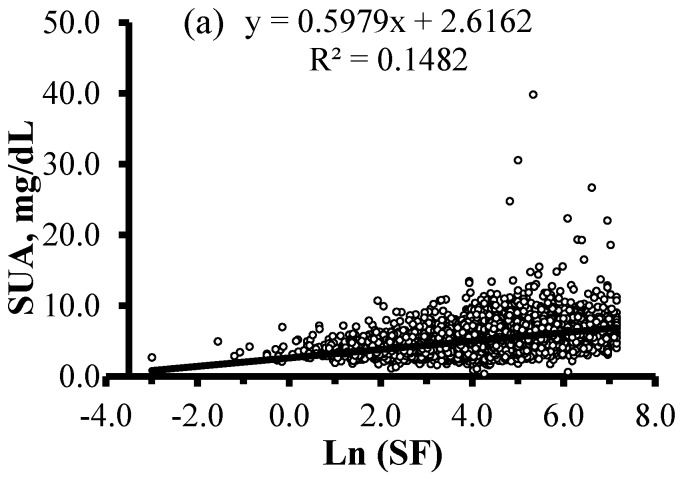

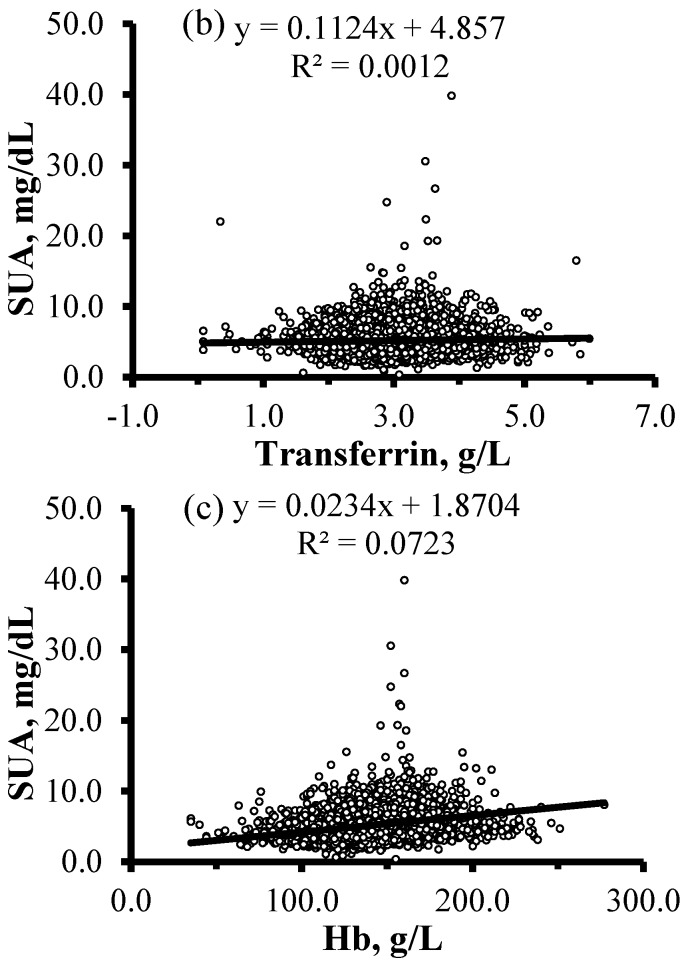
Correlations between SUA concentrations and levels of ln(SF), transferrin, and Hb in 7946 adult participants. (**a**) Positive correlation between ln(SF) and SUA (*r* = 0.385, *p* < 0.001); (**b**) positive correlation between transferrin and SUA (*r* = 0.035, *p* = 0.002); (**c**) positive correlation between Hb and SUA (*r* = 0.269, *p* < 0.001). SUA, serum uric acid; SF, serum ferritin; Hb, hemoglobin.

Figure 1 presents mean concentrations of SUA and the prevalence of HU according to age and gender. The mean concentrations of SUA were 5.2 ± 1.8 mg/dL, and male subjects had significantly higher concentrations of SUA than female participants (6.0 ± 1.9 mg/dL vs. 4.5 ± 1.3 mg/dL, *p* < 0.001). The crude prevalence of HU in the study population was 16.1% (21.4% in male and 11.4% in female). The concentrations of SUA and the prevalence of HU increased together with age in the total sample (all *p*-trend < 0.001). The concentrations of SUA decreased along with age in the male participants (*p*-trend = 0.010), whereas increased in the female participants (*p*-trend < 0.001). The prevalence of participants with HU increased in the female participants (*p*-trend < 0.001), but remained almost stable in male participants (*p*-trend = 0.531). Prior to controlling for an effect of any variable, there were significant, positive correlations of SUA with ln(SF), transferrin, and Hb (*r* = 0.385, *p* < 0.001; *r* = 0.035, *p* = 0.002; *r* = 0.269, *p* < 0.001, respectively) (Figure 2).

The concentrations of SF, transferrin, and Hb were significantly, positively associated with SUA concentrations in a dose–response manner (all *p*-trend < 0.001) after controlling for clustering of data at multiple levels (individual, household, community, and province) and adjustment for potential confounders, including age, gender, nationality, education, BMI, smoking status, alcohol consumption, and physical activity (Table 2). Those dose–response associations did not change when further adjusted for dietary intakes of total energy, protein, and fat, carbohydrate (E%), protein (E%), fat (E%), and inflammation status, or when restricted to men or women (Tables S1 and S2). Compared with participants with the lowest quartile of SF, those with the highest quartile showed significantly higher SUA concentrations (by 0.899 mg/dL, 95% CI: 0.788, 1.010; *p* < 0.001). Similarly, participants with the highest quartiles of transferrin or Hb presented significantly higher SUA concentrations by 0.488 mg/dL (95% CI: 0.389, 0.587; *p* < 0.001) or 0.353 mg/dL (95% CI: 0.235, 0.471; *p* < 0.001), respectively, when compared to those in the reference groups.

To examine the associations of SF, transferrin, and Hb with the prevalence of HU, we applied multilevel logistic regression models. The concentrations of SF and transferrin were significantly, positively associated with the prevalence of HU in a dose–response manner (all *p*-trend < 0.05) after controlling for cluster effects and adjustment for potential confounders, including age, gender, nationality, education, BMI, smoking status, alcohol consumption, and physical activity (Table 3). Those dose–response associations did not change when further adjusted for intakes of total energy, protein, and fat, carbohydrate (E%), protein (E%), fat (E%), and inflammation status, or when restricted to men or women (Tables S3 and S4). Compared with participants with the lowest quartile of SF levels, those with the highest quartile had significantly increased the risk of HU (OR: 3.086; 95% CI: 2.450, 3.888; *p* < 0.001). Similarly, compared with participants with the lowest quartile of transferrin levels, those with the highest quartile had significantly increased risk of HU (OR: 1.900; 95% CI: 1.579, 2.286; *p* < 0.001). Though significant, positive associations between Hb and the risk of HU were found in total participants and male participants (*p*-trend < 0.05), those associations were not found in female participants (*p*-trend > 0.05). Compared with participants with the lowest quartile of Hb levels, those with the highest quartile tended to have increased the risk of HU (OR: 1.218; 95% CI: 0.984, 1.507; *p* = 0.070). In male participants, those with the highest quartile of Hb levels had significantly higher risk of HU when compared to the reference group (OR: 1.401; 95% CI: 1.104, 1.777; *p* < 0.01); however, this association was not found in female participants (OR: 1.093; 95% CI: 0.821, 1.455; *p* = 0.544).

## 4. Discussion

This nationwide population-based study suggested that HU was one of the common metabolic disorders in Chinese adults, and its prevalence increased along with age, especially in female adults. It was found that ln(SF), transferrin, and Hb levels were significantly, positively correlated with SUA concentrations in our study. Furthermore, we found that levels of SF, transferrin, and Hb were positively associated with SUA concentrations and risk of HU, after controlling for cluster effects and adjustment for potential confounders. However, a significant association between Hb levels and the risk of HU was not found in female participants.

The data for this study were derived from CHNS, a large-scale longitudinal, household-based survey in China. The CHNS was designed to represent a set of large provinces which cover approximately 56% of the Chinese population, and those chosen provinces represented a range of demographic and economic variation in China [31]. The crude prevalence rate of HU (16.1% overall, 21.4% in male, and 11.4% in female) was highly comparable to the Australian population-based study (16.6% overall, 17.8% in male, and 15.4% in female) [48], and close to the 15.6% overall prevalence (20.6% in male and 13.5% in female) in the Mexican population [49]. It was lower than the longitudinal, nationwide databases of selected populations in the US (21.4% overall, 21.2% in male and 21.6% in female) [38] and Japan (25.8% overall, 34.5% in male and 11.6% in female) [50]. From a systematic review and meta-analysis [51] on relevant articles from 2000 to 2014, population-based Chinese studies reported a prevalence ranging from 7.5% to 23.6%, and the pooled prevalence of HU was 13.3% (19.4% in male and 7.9% in female), which was in accordance with our study. Our result showed that the prevalence of HU increased with advancing age, and was higher in men than women, which was consistent with many previous studies [38,48,50,52]. The analysis has shown the prevalence of HU among females was positively associated with age, whereas among males, this significant association was not found. Those gender-specific associations between HU and age agreed with findings from previous studies [50,52].

Based on existing evidence, this is the first study conducted on a large nationwide population, which aims to explore the association of SF, transferrin, and Hb levels with SUA concentrations and prevalence of HU in the Chinese population. In this study, we observed that SF levels were significantly, positively associated with SUA concentrations, and the prevalence of HU after controlling for cluster effects and adjustment for potential confounders. Such findings were consistent with several previous studies. For example, Ghio et al. [2] found that SF concentrations correlated positively with SUA concentrations after adjustment of confounding factors in 9726 healthy Americans. Mainous et al. [16] reached a similar conclusion in the same cohort that elevated levels of SUA were associated with elevated levels of SF, and might serve as a risk stratification variable for presence of iron overload. Flais et al. [15] reported that the increase in uricemia was associated with the increase in SF in 738 French patients with hemochromatosis. Zhang et al. [17] conducted a 3-year study on a large company-based cohort of Chinese employees, and found that high SF levels increased the risk of HU. The associations of dietary factors, physical activity, and hs-CRP with SUA concentrations, were reported in a substantial number of previous studies [12,13,53,54,55,56]. Thus, we constructed a series of statistical models to control the influences from the aforementioned potential confounders. Our results indicated that the positive associations of SF levels with SUA concentrations and the prevalence of HU were stable in the large-scale human study, and were independent of effects of the survey area, age, gender, BMI, race/nationality, smoking, drinking, physical activity, dietary intake, and inflammation status. Iron transport in the plasma is performed by transferrin, which transfers iron into cells through its interaction with transferrin receptors (TfR) [57]. In our study, we firstly reported that transferrin levels were associated with SUA concentrations and the risk of HU, which was involved in iron metabolism. In the present study, we found that Hb levels were significantly, positively associated with SUA concentrations and the risk of HU, in agreement with the findings from previous studies with small sample size [18,58].

The details of the underlying mechanism about the effects of SF, transferrin, and Hb on SUA remain to be determined. Ferritin can convert Fe^2+^ to Fe^3+^, due to its high iron-binding capacity and ferroxidase activity. The majority of intracellular iron is stored in ferritin as a compact, safe way to prevent the toxic effects of iron and make iron available when necessary; therefore, SF is widely used to evaluate iron status in clinical practice [59]. About 70% of the body’s iron is found in red blood cells, which constitutes the crucial part of Hb. SUA, as a major antioxidant in human blood, can form a 2:1 complex with Fe^3+^ ions to inhibit iron-catalyzed oxidations [60,61]. The relationships of SF and Hb with HU could be a response to oxidative stress linked with the visceral toxicity of excess non-transferrin bound iron via the antioxidant properties of SUA [15]. Xanthine oxidase (XO), one of the key purine metabolism enzymes responsible for producing SUA, may be involved in this process. Elevated expression and activity of XO following exposure to iron was observed in both in vivo and in vitro studies [62,63,64]. On the contrary, lung XO and total xanthine oxidoreductase activities were reduced in rats fed an iron-depleted diet [64]. The production of pro-inflammatory cytokines (including TNF-α, IL-1, and IL-6) secondary to oxidative stress induced by iron overload, could also take part in increasing XO activity [65]. Another underlying mechanism of the relationship between SF and SUA might be due to insulin sensitivity. Accumulating evidence suggests that elevated SF levels is associated with higher fasting insulin levels, insulin resistance, and an increased risk of diabetes [66,67,68]. Contrarily, a protective factor for the development of diabetes was observed in healthy people with frequent blood donation, leading to decreased iron stores [69]. Higher insulin levels and insulin resistance can reduce renal excretion of urate, and contribute to increase uric acid synthesis [70,71,72]. Therefore, the link between SF and insulin sensitivity may be translated into an independent association between elevated SF and the pathogenesis of HU.

There are several limitations in our study. First, with regard to the cross-sectional nature, it is not appropriate to establish a causal relationship in our study, and the temporal effects of SF, transferrin, and Hb on the development of HU remains to be understood. Second, even though numbers of confounders were adjusted, we cannot rule out the possibility that unmeasured factors or residual confounding might contribute to the observed associations given the nature of our observational study. The third limitation was the lack of information on the menopausal status, but a soaring increase of HU in female aged 50 years or over was observed in our study.

## 5. Conclusions

Our findings indicated that SF, transferrin, and Hb were positively associated with SUA concentrations and the prevalence of HU in the Chinese adult population. Additional studies are needed to confirm these findings, as well as to elucidate their underlying mechanisms. To prevent or delay the progression of gout and some other NCDs, substantial attention has been devoted in recent years to controlling their potentially modifiable risk factors. Thus, further studies are warranted to determine whether SF, transferrin, and Hb reduction may improve the HU risk factors, and thereby reduce the risk of developing gout, diabetes mellitus, cardiovascular diseases, nonalcoholic fatty liver disease, and cancer.

## Figures and Tables

**Table 1:** Characteristics of the study participants from the China Health and Nutrition Survey in 2009.

Characteristics	HU (*n* = 1276)	Non-HU (*n* = 6670)	Total (*n* = 7946)	*p*
SUA, mg/dL	7.9 ± 2.1	4.7 ± 1.1	5.2 ± 1.8	<0.001
Rural area				<0.001
Yes	785 (61.5)	4546 (68.2)	5331 (67.1)	
No	491 (38.5)	2124 (31.8)	2615 (32.9)	
Age, years	53.4 ± 15.2	50.3 ± 14.8	50.8 ± 14.9	<0.001
Gender				<0.001
Male	793 (62.1)	2917 (43.7)	3710 (46.7)	
Female	483 (37.9)	3753 (56.3)	4236 (53.3)	
Nationality				0.205
Han	1123 (88.0)	5951 (89.2)	7074 (89.0)	
Others	153 (12.0)	719 (10.8)	872 (11.0)	
Education, years	9 (5, 12)	9 (5, 9)	9 (5, 9)	0.032
BMI, kg/m^2^	24.8 ± 3.6	23.1 ± 3.4	23.4 ± 3.5	<0.001
Current smoker				<0.001
Yes	438 (34.3)	1759 (26.4)	2197 (27.6)	
No	838 (65.7)	4911 (73.6)	5749 (72.4)	
Alcohol consumption				<0.001
Yes	560 (43.9)	2047 (30.7)	2607 (32.8)	
No	716 (56.1)	4623 (69.3)	5339 (67.2)	
Physical activities, MET-h/week	73.6 (31.5, 147.4)	87.3 (37.0, 182.0)	84.5 (35.9, 177.0)	<0.001
Total energy intake, kcal/day	2167.4 (1772.0, 2580.1)	2063.1 (1669.1, 2499.2)	2081.6 (1680.7, 2512.3)	<0.001
Carbohydrate intake, g/day	281.4 (219.3, 354.2)	282.4 (224.2, 354.8)	282.3 (223.4, 354.7)	0.860
Carbohydrate (E%)	54.2 ± 10.7	56.3 ± 10.7	55.9 ± 10.7	<0.001
Protein intake, g/day	66.0 (52.9, 82.3)	61.9 (49.6, 77.8)	62.6 (50.1, 78.6)	<0.001
Protein (E%)	12.9 ± 3.0	12.6 ± 2.9	12.6 ± 2.9	<0.001
Fat intake, g/day	75.7 (54.2, 99.2)	68.3 (48.7, 93.1)	69.5 (49.5, 94.4)	<0.001
Fat (E%)	32.9 ± 10.8	31.2 ± 10.7	31.4 ± 10.7	<0.001
SF, μg/L	119.3 (71.0, 212.4)	71.1 (34.9, 131.0)	78.0 (39.4, 143.3)	<0.001
Transferrin, g/L	3.0 ± 0.6	2.9 ± 0.5	2.9 ± 0.5	<0.001
sTfR, mg/L	1.33 (1.07, 1.68)	1.34 (1.09, 1.65)	1.34 (1.09, 1.65)	0.407
Hb, g/L	146.3 ± 20.9	140.6 ± 20.3	141.5 ± 20.5	<0.001
Hs-CRP, mg/L	2.0 (1.0, 3.0)	1.0 (0.0, 2.0)	1.0 (0.0, 2.0)	<0.001

HU, hyperuricemia; SUA, serum uric acid; BMI, body mass index; MET-h, metabolic equivalent hours; E%, percentage of energy; SF, serum ferritin; sTfR, soluble transferrin receptor; Hs-CRP, high-sensitivity C-reactive protein; Hb, hemoglobin. Data were expressed as means ± standard deviation or medians (interquartile ranges) for continuous variables with or without normal distribution respectively and counts (percentages) for categorical variables. Comparisons between participants with hyperuricemia and the other participants were performed by using Student’s *t* tests, Mann–Whitney U tests, or Chi-squared tests.

**Table 2:** Multilevel-adjusted associations of SF, transferrin, and Hb levels with SUA concentrations in Chinese adult population (*n* = 7946).

		SUA, mg/dL	β (95% CI) ^1^	Adjust β (95% CI) ^2^	Adjust β (95% CI) ^3^
SF	Q1	4.26 ± 1.25	Ref.	Ref.	Ref.
	Q2	4.91 ± 1.46	0.658 (0.555, 0.761)	0.310 (0.209, 0.411)	0.267 (0.167, 0.367)
	Q3	5.46 ± 1.57	1.202 (1.099, 1.305)	0.587 (0.481, 0.693)	0.529 (0.423, 0.635)
	Q4	6.10 ± 2.19	1.842 (1.739, 1.945)	0.991 (0.879, 1.103)	0.899 (0.788, 1.010)
	*p*-trend	<0.001	<0.001	<0.001	<0.001
Transferrin	Q1	5.05 ± 1.54	Ref.	Ref.	Ref.
	Q2	5.15 ± 1.61	0.094 (−0.017, 0.205)	0.196 (0.098, 0.294)	0.208 (0.112, 0.304)
	Q3	5.27 ± 1.73	0.216 (0.105, 0.327)	0.334 (0.235, 0.433)	0.351 (0.254, 0.448)
	Q4	5.26 ± 2.19	0.209 (0.098, 0.320)	0.457 (0.357, 0.557)	0.488 (0.389, 0.587)
	*p*-trend	<0.001	<0.001	<0.001	<0.001
Hb	Q1	4.58 ± 1.50	Ref.	Ref.	Ref.
	Q2	4.79 ± 1.47	0.213 (0.109, 0.317)	0.046 (−0.051, 0.143)	0.029 (−0.066, 0.124)
	Q3	5.45 ± 1.73	0.876 (0.769, 0.983)	0.148 (0.039, 0.257)	0.114 (0.006, 0.222)
	Q4	5.98 ± 2.08	1.402 (1.294, 1.510)	0.388 (0.269, 0.507)	0.353 (0.235, 0.471)
	*p*-trend	<0.001	<0.001	<0.001	<0.001

SUA, serum uric acid; CI, confidence interval; SF, serum ferritin; Ref., reference; Hb, hemoglobin; BMI, body mass index; E%, percentage of energy; Hs-CRP, high-sensitivity C-reactive protein. All of the models were constructed by using multilevel (four levels) mixed-effects linear regression with the iterative generalized least-squares method. The medians (interquartile ranges) or means ± standard deviation for quartiles of SF, transferrin, and Hb were as follows—SF: 20.3 (11.3, 29.3), 58.0 (48.7, 67.8), 104.5 (90.7, 121.7), and 237.8 (174.6, 446.5) μg/L; transferrin: 2.3 ± 0.3, 2.7 ± 0.1, 3.0 ± 0.1, 3.6 ± 0.4 g/L; Hb: 116.9 ± 11.8, 135.0 ± 3.8, 147.8 ± 3.7, 167.8 ± 13.9 g/L. ^1^ Original model without any adjustments. ^2^ Adjusted for age, gender (male or female), nationality (Han or others), education (≤6 years, 6.1–9.0 years, or >9 years), smoking status (current or not current), alcohol consumption (yes or no), BMI (<18.5 kg/m^2^, 18.5–24.9 kg/m^2^, 25.0–29.9 kg/m^2^, or ≥30 kg/m^2^), and physical activity (quartile). ^3^ Adjusted as for model 2 plus total energy intake (quartile), protein intake (quartile), fat intake (quartile), carbohydrate (E%) (quartile), protein (E%) (quartile), fat (E%) (quartile), and inflammation status (hs-CRP levels < 1 mg/L, 1–3 mg/L or 4–10 mg/L).

**Table 3:** Multilevel-adjusted associations of SF, transferrin, and Hb levels with the risk of HU in Chinese adult population (*n* = 7946).

		Cases/Total, *n*	β (95% CI) ^1^	Adjust OR (95% CI) ^2^	Adjust OR (95% CI) ^3^
SF	Q1	126/1987	Ref.	Ref.	Ref.
	Q2	248/1987	2.106 (1.684, 2.635)	1.726 (1.368, 2.179)	1.618 (1.280, 2.044)
	Q3	376/1987	3.449 (2.790, 4.263)	2.550 (2.030, 3.203)	2.344 (1.863, 2.951)
	Q4	526/1985	5.323 (4.332, 6.540)	3.487 (2.773, 4.384)	3.086 (2.450, 3.888)
	*p*-trend	<0.001	<0.001	<0.001	<0.001
Transferrin	Q1	261/2016	Ref.	Ref.	Ref.
	Q2	307/1991	1.226 (1.026, 1.465)	1.298 (1.080, 1.560)	1.318 (1.094, 1.587)
	Q3	333/1953	1.383 (1.160, 1.649)	1.496 (1.246, 1.797)	1.539 (1.279, 1.852)
	Q4	375/1986	1.565 (1.318, 1.859)	1.817 (1.514, 2.179)	1.900 (1.579, 2.286)
	*p*-trend	<0.001	<0.001	<0.001	<0.001
Hb	Q1	237/1949	Ref.	Ref.	Ref.
	Q2	277/2182	1.050 (0.873, 1.264)	0.948 (0.784, 1.147)	0.918 (0.757, 1.113)
	Q3	336/1924	1.528 (1.276, 1.829)	1.054 (0.862, 1.289)	0.996 (0.812, 1.221)
	Q4	426/1891	2.100 (1.766, 2.497)	1.288 (1.044, 1.589)	1.218 (0.984, 1.507)
	*p*-trend	<0.001	<0.001	0.008	0.036

HU, hyperuricemia; SF, serum ferritin; CI, confidence interval; OR, odds ratio; Hb, hemoglobin; Ref., reference; BMI, body mass index; E%, percentage of energy; Hs-CRP, high-sensitivity C-reactive protein. All of the models were constructed by using multilevel (four levels) mixed-effects logistic regression with the restricted iterative generalized least-squares estimation method. The medians (interquartile ranges) or means ± standard deviation for quartiles of SF, transferrin, and Hb were as follows—SF: 20.3 (11.3, 29.3), 58.0 (48.7, 67.8), 104.5 (90.7, 121.7), and 237.8 (174.6, 446.5) μg/L; transferrin: 2.3 ± 0.3, 2.7 ± 0.1, 3.0 ± 0.1, 3.6 ± 0.4 g/L; Hb: 116.9 ± 11.8, 135.0 ± 3.8, 147.8 ± 3.7, 167.8 ± 13.9 g/L. ^1^ Original model without any adjustments. ^2^ Adjusted for age, gender (male or female), nationality (Han or others), education (≤6 years, 6.1–9.0 years, or >9 years), smoking status (current or not current), alcohol consumption (yes or no), BMI (<18.5 kg/m^2^, 18.5–24.9 kg/m^2^, 25.0–29.9 kg/m^2^, or ≥30 kg/m^2^), and physical activity (quartile). ^3^ Adjusted as for model 2 plus total energy intake (quartile), protein intake (quartile), fat intake (quartile), carbohydrate (E%) (quartile), protein (E%) (quartile), fat (E%) (quartile), and inflammation status (hs-CRP levels < 1 mg/L, 1–3 mg/L or 4–10 mg/L).

## Supplementary Materials

The following are available online at http://www.mdpi.com/2072-6643/10/2/191/s1, Table S1: Multilevel-adjusted associations of SF, transferrin, and Hb levels with SUA concentrations in male adult population (*n* = 3710), Table S2: Multilevel-adjusted associations of SF, transferrin, and Hb levels with SUA concentrations in female adult population (*n* = 4236), Table S3: Multilevel-adjusted associations of SF, transferrin, and Hb levels with the risk of HU in male adult population (*n* = 3710), Table S4: Multilevel-adjusted associations of SF, transferrin, and Hb with the risk of HU in female adult population (*n* = 4236).

## Abbreviations

BMI	body mass index
CHNS	the China Health and Nutrition Survey
CI	confidence interval
CRP	C-reactive protein
E%	percentage of energy
Hb	hemoglobin
hs-CRP	high-sensitivity C-reactive protein
HU	hyperuricemia
IL-6	interleukin-6
MET	metabolic equivalent
NCDs	non-communicable diseases
OR	odds ratio
Ref.	reference
SD	standard deviation
SE	standard error
SF	serum ferritin
sTfR	soluble transferrin receptors
SUA	serum uric acid
TfR	transferrin receptors
TNF-α	tumor necrosis factor alpha
WBC	white blood cell count
XO	xanthine oxidase
